# Human liver stem cells and derived extracellular vesicles protect from sepsis-induced acute lung injury and restore bone marrow myelopoiesis in a murine model of sepsis

**DOI:** 10.1186/s40635-024-00701-z

**Published:** 2024-12-03

**Authors:** Andrea Costamagna, Chiara Pasquino, Sara Lamorte, Victor Navarro-Tableros, Luisa Delsedime, Vito Fanelli, Giovanni Camussi, Lorenzo Del Sorbo

**Affiliations:** 1 Department of Surgical Sciences, University of Turin, Turin, Italy; 2 Molecular Biotechnology Center, University of Turin, Turin, Italy; 3 Princess Margaret Cancer Center, University Health Network, Toronto, ON Canada; 4 2i3T – Scarl.-Molecular Biotechnology Center (MBC), University of Turin, Turin, Italy; 5 Pathology Unit, A.O.U, Città Della Salute E Della Scienza Di Torino, Turin, Italy; 6 Department of Medical Sciences, University of Turin, Turin, Italy; 7 Interdepartmental Division of Critical Care Medicine, University Health Network, University of Toronto, Toronto, ON Canada

**Keywords:** Shock, Septic, Mesenchymal stem cells, Extracellular vesicles, Acute lung injury, Myelopoiesis

## Abstract

**Background:**

Sepsis is a condition with high mortality and morbidity, characterized by deregulation of the immune response against the pathogen. Current treatment strategies rely mainly on antibiotics and supportive care. However, there is growing interest in exploring cell-based therapies as complementary approaches. Human liver stem cells (HLSCs) are pluripotent cells of mesenchymal origin, showing some advantages compared to mesenchymal stem cells in terms of immunomodulatory properties. HSLC-derived extracellular vesicles (EVs) exhibited a superior efficacy profile compared to cells due to their potential to get through biological barriers and possibly to avoid tumorigenicity and showed to be effective in vivo and ex vivo models of liver and kidney disease. The potential of HLSCs and their EVs in recovering damage to distal organs due to sepsis other than the kidney remains unknown. This study aimed to investigate the therapeutic potential of the intravenous administration of HSLCs or HSLCs-derived EVs in a murine model of sepsis.

**Results:**

Sepsis was induced by caecal ligation and puncture (CLP) on C57/BL6 mice. After CLP, mice were assigned to receive either normal saline, HLSCs or their EVs and compared to a sham group which underwent only laparotomy. Survival, persistence of bacteraemia, lung function evaluation, histology and bone marrow analysis were performed. Administration of HLSCs or HLSC-EVs resulted in improved bacterial clearance and lung function in terms of lung elastance and oedema. Naïve murine hematopoietic progenitors in bone marrow were enhanced after treatment as well. Administration of HLSCs and HLSC-EVs after CLP to significantly improved survival.

**Conclusions:**

Treatment with HLSCs or HLSC-derived EVs was effective in improving acute lung injury, dysmyelopoiesis and ultimately survival in this experimental murine model of lethal sepsis.

**Supplementary Information:**

The online version contains supplementary material available at 10.1186/s40635-024-00701-z.

## Background

Sepsis is a common condition with a high mortality rate, which reaches over 40% in the most severe cases of septic shock [1–3]. A body of evidence indicates that during sepsis the deregulation of the immune response against the pathogen results in multiorgan dysfunctions [4], including acute respiratory distress syndrome (ARDS) [5, 6], acute kidney injury and bone marrow derangement with thrombocytopenia [7] and myelopoiesis impairment [8]. Currently, treatment for sepsis remains timely and appropriate antimicrobial therapy, effective infection source control and supportive care [9–12].

The effects and mechanisms of cell-based therapy [5, 13] have been investigated in sepsis. In several pre-clinical models of sepsis, mesenchymal stromal cells (MSCs) have been shown to reduce inflammation, and enhance bacterial clearance and recovery from lung injury [5, 14–16]. In addition, MSCs have already been tested in phase 1 clinical trial in patients with septic shock, showing good tolerance and lack of significant adverse events [17, 18]. However, the use of MSCs in treating sepsis raised several significant concerns. Schlosser et al. found that MSCs elicited a temporary increase in pro-inflammatory cytokines, such as IL-6, IL-8 and MCP-1, when given at high doses [18]. HLA mismatched MSCs are susceptible to natural killer (NK) mediated cytotoxicity [19], which has been consistently shown to be the leading cause of MSC death [20].

Extracellular vesicles (EVs) represent a varied group of particles originating in the cytosol and capable of being secreted by various cell types. EVs are composed of a membrane-enclosed cargo that mediates cellular communication upon release into the extracellular space [21]. In different pre-clinical disease models, MSC-released EVs (MSC-EVs) showed the same therapeutic effect compared to the entire cell but with fewer adverse effects (i.e., absence of allogenic-driven immune response and theoretical oncogenic potential). Moreover, they offer distinct advantages such as higher suitability for long-term storage and direct mRNA-mediated cell-to-cell communication, with stronger signalling [22].

Human liver stem cells (HLSCs) have been isolated in 2006 [23]. These cells are pluripotent and even if they do not express hematopoietic markers, they share some identity phenotype with MSCs, disclosing a mesenchymal origin together with immunomodulatory properties [24]. These cells were able to promote reduction of liver fibrosis and inflammation by reducing mRNA expression of Il-1β and Ifn-γ, as well as leukocyte infiltration, in a murine model of non-alcoholic steatohepatitis. [23, 25, 26]. In addition, HLSC-EVs were effective in reducing phospho-mTOR, a key downstream target of the PI3K/Akt/mTOR pathway, suggesting potential anti-inflammatory activity. [27] PI3Kγ is known to play a critical role in neutrophil recruitment, and its inhibition reduced mortality and organ damage in a murine model of CLP-induced sepsis. [28] However, the therapeutic potential of HLSCs in recovering organs other than the liver has only been shown in a mouse model of acute kidney injury (AKI) [29, 30]. The potential of HLSCs and their EVs in recovering distal organs other than the kidney and the liver remains unknown.

HSLCs showed some theoretical advantages compared to MSCs regarding the immunomodulatory properties in an allogenic in vitro setting. In particular, HSLCs were able to inhibit *T*-cell proliferation to a greater extent than MSCs when co-cultured with allogenic CD3^+^ cells [31] Moreover, the incubation of natural killer cells (NK) with HSLCs did not induce NK degranulation, as in contrast to MSCs [31]. In addition, HSLCs demonstrated to be effective in inhibiting *T*-cells proliferation at lower doses compared to MSCs [24, 31].

Extracellular vesicles derived from HSLCs have recently been characterized. They exhibit a superior efficacy profile compared to cells, essentially due to their potential to get through biological barriers and possibly to avoid tumorigenicity. They showed to be effective in in vivo and ex vivo models of liver and kidney disease [24, 29].

The aim of this work was to investigate the therapeutic effect of the intravenous administration of HSLCs or HSLC-derived extracellular vesicles in a clinically relevant murine model of sepsis.

Our hypothesis was that treatment with HLSCs or HLSC-EVs could improve acute lung injury and dysmyelopoiesis and ultimately enhance survival.

## Methods

### Animals

C57/BL6 male mice weighing 25–30 g were purchased from Charles Rivers (Italy). The experimental protocol (number 0253358) for this study was in accordance with the institutional animal welfare guidelines and approved by the Bioethical Committee of the University of Turin (approval date: 23/02/2012). Mice were housed, provided free access to standard food and water in a controlled facility with a 12-h light and dark cycle.

### Culture of HLSC and fibroblasts

HLSCs were isolated from human cryopreserved normal hepatocytes provided in kind by Lonza (Basel, Switzerland) and characterized as previously described (Herrera et al., 2006). Briefly, HLSCs were cultured in a standard culture medium containing a 3 to 1 proportion of α-minimum essential medium and endothelial cell basal medium-1, supplemented with L-glutamine 2 mM, penicillin 100 UI/ml/streptomycin 100 µg/ml and 10% Fetal Calf Serum (α-MEM/EBM/FCS; Gibco/Cambrex) and maintained in a humidified 5% CO2 incubator at 37 °C. Cells at about 80% confluence were trypsinized and harvested by centrifugation at 1200 rpm for 5 min and used for further expansion or for in vivo injection.

Primary normal Human Dermal Fibroblasts (NHDF) (provided in kind by Lonza, Basel, Switzerland) were cultured in FGM-2 Growth Media (Lonza, Basel, Switzerland) supplemented with bullet kit. Cells at about 80% confluence were trypsinized and harvested by centrifugation at 1200 rpm for 5 min and used for further expansion or for in vivo injection.

### Isolation and characterization of EVs from HLSCs

EVs were obtained from supernatants of HLSCs cultured in RPMI medium (Fig. S1). After centrifugation at 3000* g* for 20 min to remove debris, followed by a filtration using a 0.22 μm vacuum filter unit to eliminate large vesicles, the cell-free supernatants were centrifuged at 100000* g* (Beckman Coulter Optima *L*-90 K ultracentrifuge; Beckman Coulter, Fullerton, CA) for 2 h at 4 °C. EVs were resuspended in RPMI + 1% DMSO and stored at − 80 °C. Each EV preparation was verified by size distribution using the NanoSight NS300 system (NanoSight, Amesbury, UK): 5 different preparation were diluted in sterile saline solution (1:200) and particle number and size were analysed with NanoParticle Tracking Analysis (NTA) System of the NTA 3.2 Analytical Software as described previously [32].

EV phenotype was confirmed using bead-based multiplex analysis by flow cytometry (MACSPlex Exosome Kit, human, Miltenyi Biotec) [33]. Briefly, 1 × 10^9^ EVs were diluted in 120 μL of MACSPlex buffer (MPB) and 15 μL of MACSPlex Exosome Capture Beads (containing 39 different antibody-coated bead subsets) and 5 μL each of APC-conjugated anti-CD9, anti-CD63, and anti-CD81 detection antibodies were added to each tube. The suspensions were then incubated in an orbital shaker for 1 h at 450 rpm at room temperature, protected from light. After washing, the supernatant was carefully removed and EVs were used for the acquisition using BD FACSCelesta™ Flow Cytometer. The analysis of the marker expression was performed using the BD FACSDiva™ Software.

Approximately 5000 single-bead events per sample have been recorded. Median fluorescence intensity (MFI) for all 39 capture bead subsets was subjected to a correction of background by subtracting respective MFI values from matched media controls. All bead populations can be identified and gated based on their respective fluorescence intensity according to the manufacturer’s instructions.

### Sepsis model caused by caecal ligation and puncture

**Fig. 1:**
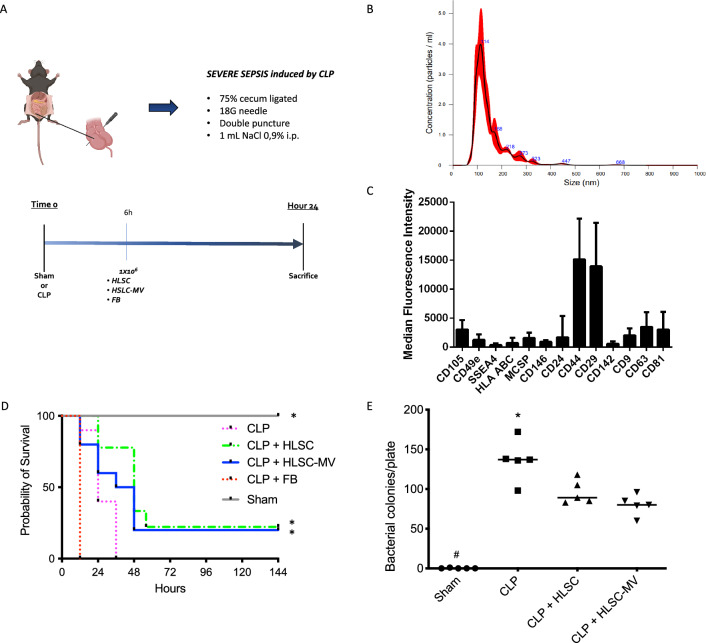
**Panel A**: In-vivo experimental model of sepsis. The figure represents the caecal ligation and puncture (CLP) procedure together with the experimental protocol timeline. **Panel B:** HLSC-EVs Characterization. Representative Nanoparticle tracking analyses showing the size distribution of HLSC-EVs, with scale bar of 100 nm. **Panel C:** MACS multiplex bead-based flow cytometry assay of different surface markers, only expressed markers were reported as mean fluorescent intensity (MFI). The graph shows a quantification of the median APC fluorescence values for all bead populations after background correction (medium control values subtracted from measured EV-HLSC values) of five different preparations. No statistically significant differences were observed among them. **Panel D:** Survival analysis among groups (Gehan–Breslow–Wilcoxon test). CLP: mice subjected to Caecal Ligation and Puncture treated with IV normal saline; HLSCs: mice subjected to Caecal Ligation and Puncture treated with IV Human Liver Stem Cells; HLSC-EVs: mice subjected to Caecal Ligation and Puncture treated with IV Human Liver Stem Cells’ Extracellular vesicles; FB: mice subjected to Caecal Ligation and Puncture treated with IV Fibroblasts. * = *p* < 0.001 versus CLP and FB groups. **Panel E:** Analysis of the bacterial load in peripheral blood samples. Sham: mice subjected to simple median laparotomy without performing CLP; CLP: mice subjected to Caecal Ligation and Puncture treated with IV normal saline; HLSCs: mice subjected to Caecal Ligation and Puncture treated with IV Human Liver Stem Cells; HLSC-EVs: mice subjected to Caecal Ligation and Puncture treated with IV Human Liver Stem Cells’ Extracellular vesicles. *p < 0.05 CLP versus all other groups; ^#^*p* < 0.05 Sham versus all other groups. One-way ANOVA with Tukey post-hoc test

The caecal ligation and puncture (CLP) model (Fig. 1a) was performed as previously described [34]. Briefly, after a ventral midline incision (1 cm), the cecum was ligated by silk 7–0 immediately after the valve to induce high-grade sepsis, then the cecum was punctured twice through-and-through with a 18-gauge needle and returned into the abdominal cavity. In sham-operated mice, the cecum was exteriorized and manipulated but not ligated or punctured. After surgical suture, 1 ml NaCl 0.9% was administered intra-peritoneally. After the surgical procedure, an of 1 mg/Kg of tramadol chlorhydrate was injected intramuscularly at 0, 6, 24 and 48 h, as a postoperative analgesia; no antibacterial therapy was administrated.

After CLP, three groups of animals were randomly assigned to receive through the tail vein either 0.2 ml normal saline (CLP group, *n* = 5) as positive control, 1*10^6^ Human Liver Stem Cells resuspended in 0.2 ml normal saline (HSLC group, *n* = 5) or 1.4*10^9^ of Human Liver Stem Cells’ Extracellular vesicles resuspended in 0.2 ml normal saline (HSLC-EV group, *n* = 5). HSLC and HSLC-EV dose was chosen based on previous published data and unpublished pilot experiments. [29, 35] Randomization was performed using opaque sealed envelopes containing the randomization schedule. Injection in the tail vein was performed always by the same operator (VN), who was the only investigator unblinded and was not involved in the assessment of experimental outcomes. A fourth group which underwent laparotomy without CLP was obtained as negative control group (SHAM group, *n* = 5). Animals were euthanized under anaesthesia by exsanguination via cardiac puncture with subsequent cervical dislocation.

### Survival

In a separate experiment, mice from each experimental group (*n* = 9 per group) were monitored for survival during the six postoperative days (144 h). This study included a fifth experimental group, which received 1*10^6^ NHDFs intravenously after CLP (FB group).

### Lung mechanics

Twenty-four hours after CLP or sham surgery, mice were anaesthetised, the trachea was exposed after median neck incision and a tracheostomy with a 20G cannula (standard length) was performed to allow measurement of lung mechanical properties. Full median sternotomy was then performed with mediastinum exposure, and full blood was collected from direct cardiac puncture.

Lungs were ventilated through the tracheostomy tube to derive lung elastance and airway pressure; gas flow was recorded (ICU-Lab, KleisTEK Advanced Electronic Systems, Bari, Italy). The diaphragm was removed and a median sternotomy with ribs’ draw back was performed to eliminate the chest wall components of elastance. Lung elastance was calculated with the super syringe method [36]: briefly, a 30 ml syringe linked to a mechanical pump with electronic controls was connected to the tracheostomy tube after a few seconds of disconnection of the lungs from the system to allow complete lung deflation. The airway pressure was measured by a pressure transducer (zero referred to atmospheric pressure). Gas flow was constant at 2 ml/min and the inflation was stopped once reached an inspiratory pressure (Pi) of 30 cmH_2_O. Positive end-expiratory pressure (PEEP) was 0 cmH_2_O, so the lung elastance was calculated as follows:

$$E = \frac{\Delta P}{V} = \frac{{\left( {Pi - PEEP} \right)}}{V} = \frac{30}{V}$$

where E is lung elastance (expressed in cmH_2_O/ml), Pi is the given target maximum inspiratory pressure and V is the total volume required to reach the target Pi from the beginning of lung inflation.

After lung mechanics assessment, the following procedures were performed to assess additional endpoints.

### Broncho-alveolar lavage protein concentration and cytokines

Broncho-alveolar lavage (BAL) was performed on the isolated left lung. Briefly, left lung was lavaged twice with 0.5 ml normal saline and centrifuged at 3000 g for 10 min; the supernatant was snap-frozen and stored at − 80 °C for further analysis. BAL protein concentration was used to assess the alveolar–capillary barrier permeability. Total proteins in the BAL were measured by micro-bicinchoninic acid protein assay (Thermo Scientific, Rockford, IL).

Quantitative analysis of tumour necrosis factor-α (TNF-α) and Interleukin-10 (IL-10) in the BAL supernatant was performed using the murine and human ELISA (RayBiotech, Inc Norcross, GA, USA). Briefly, 50 µl of sample in duplicate were used and incubated following the vendor instructions, to induce a colorimetric reaction, red on the Biorad 680 microplate reader at 450 nm.

### Histological analysis

The right lung was fixed in 4% paraformaldehyde solution according to standard methods (Sigma-Aldrich) and paraffined. Lung Sects. (3 μm) were stained with haematoxylin and eosin. A pathologist blinded to experimental groups analyzed nine random fields of view. Lung injury was quantified using a 4-point score (LIS: 0, none; 1, mild; 2, moderate; 3, severe) based on four features: (1) alveolar oedema, (2) capillary congestion, (3) neutrophil infiltration and (4) septal thickening [37]

### Bacterial count in peripheral blood

Bacterial count was assessed as previously described [38]. Briefly, following sham or CLP surgery, blood was collected under sterile conditions and 10 μl of blood was plated on Muller–Hinton agar dishes (Difco Laboratories, Detroit, USA) and incubated at 37 °C. Colony-forming units (CFU) were analyzed after 24 h, and the results were expressed as CFU per ml of blood.

### Bone marrow analysis

Bone marrow was harvested from femoral and tibial diaphysis through flushing with ice-cold HBSS followed by mechanical dissociation with a plunger on a 70 μm cell strainer to obtain a mononuclear cell suspension for downstream analysis.

Colony-forming cells (CFC) were assessed using the assay based on methylcellulose medium (MethoCult™, Stem Cell Technology). The bone marrow-derived cells were cultured by plating 1 × 10^4^ cells in a 100 μm diameter in the presence of erythropoietin (10 ng/ml) for 6–7 days. The colonies formed were then counted.

For cytological analysis, bone marrow smears were stained with May–Grünwald solution. In each mouse bone marrow and blood were analyzed by flow cytometry to evaluate the frequency of hemopoietic stem cells (Sca1^+^KDR^−^ and Sca1^+^LIN^−^, respectively).

### Statistical analysis

Data are expressed as means ± standard deviation (SD). The Kolmogorov–Smirnov test confirmed that the data followed a normal distribution for each variable analysed. One-way ANOVA with Tukey post-hoc analysis was used to compare data between study groups. Kaplan–Meier analysis has been performed to assess survival with Gehan–Breslow–Wilcoxon test. Differences were considered statistically significant for *p* < 0.05. Statistical analysis was performed with Prism 9.4 software (GraphPad Software, San Diego, CA, USA).

## Results

### Characterization of HLSC-EVs

NanoSight analysis showed that HLSC-EVs have a consistent profile among different preparations, with a mean size distribution of 149 ± 25 nm (Fig. 1b). The analysis using MACS multiplex bead-based flow cytometry assay showed that the EVs expressed typical HLSC markers, such as CD44, CD105, CD146, SSEA4, CD49e and CD29, and were positive for exosomal tetraspanins (CD81, CD63, CD9) (Fig. 1c), as previously shown [35].

### Survival analysis

The administration of HLSCs and HLSC-EVs after CLP showed to significantly improve survival in the experimental animals compared to both positive control groups, who underwent CLP without further treatment (CLP group) or received 1*10^6^ NHDFs after CLP (FB group; *p* < 0.05 HLSC and HLSC-EVs vs. both CLP and FB group—Fig. 1d).

### Bacterial count in peripheral blood

In the HLSC and HLSC-EV groups, the number of bacterial CFU in the blood was significantly lower than those observed in the positive CLP control (90.5 ± 10.0 vs. 80.0 ± 15.1 vs. 135.7 ± 37.0 CFU respectively, *p* < 0.05) and comparable to those observed in the SHAM (0.2 ± 0.5 CFU) group (Fig. 1e).

### Lung mechanics

**Fig. 2:**
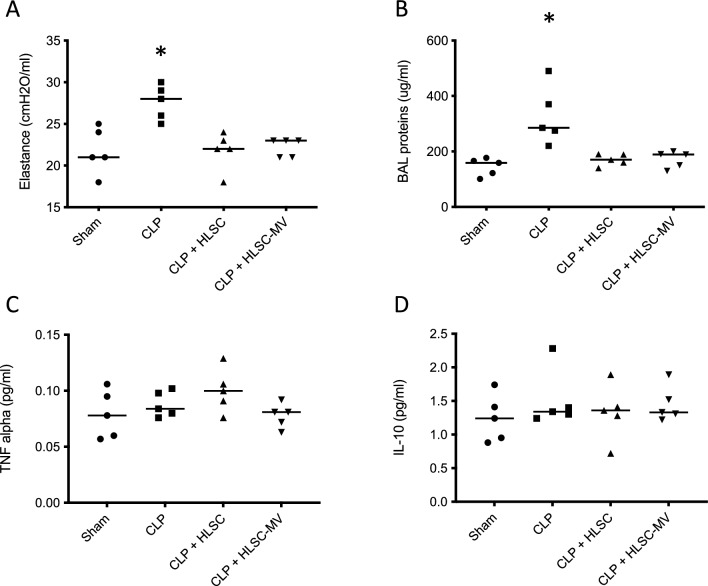
Lung specific elastance calculated with the super syringe method (**panel A**); Bronchoalveolar lavage fluid (BAL) protein concentration (**panel B**), as a surrogate of alveolar–capillary barrier permeability; Quantitative analyses of TNFα (**Panel C**) and IL10 (**panel D**) concentrations in the BAL supernatant. Sham: mice subjected to simple median laparotomy without performing CLP; CLP: mice subjected to Caecal Ligation and Puncture treated with IV normal saline; HLSCs: mice subjected to Caecal Ligation and Puncture treated with IV Human Liver Stem Cells; HLSC-EVs: mice subjected to Caecal Ligation and Puncture, treated with IV Human Liver Stem Cells’ Extracellular vesicles. *p < 0.05 CLP versus all other groups. One way ANOVA with Tukey post-hoc test

Lung elastance was significantly lower in the HLSC and HLSC-EV groups compared to the CLP group (23.1 ± 3.0 cmH_2_O/ml and 22.7 ± 1.0 cmH_2_O/ml vs. 27.1 ± 2.3 cmH_2_O/ml, respectively; *p* < 0.05) and comparable to the SHAM group (Fig. 2a).

### BAL protein concentration and cytokines

The protein concentration in the BAL, which is a surrogate measure of the alveolar–capillary permeability, was significantly lower in the HLSC and HLSC-EV groups than in the CLP group (171 ± 20 vs. 173 ± 31 vs. 326 ± 121 µg/ml, respectively; *p* < 0.05), and comparable to the SHAM group (143 ± 29 µg/ml) (Fig. 2b).

The concentration of the pro-inflammatory cytokine TNFα and the anti-inflammatory cytokine IL-10 was not affected by CLP (TNFα 0.09 ± 0.01 and IL-10 1.50 ± 0.44 pg/ml), HSLCs (TNFα 0.10 ± 0.02 and IL-10 1.36 ± 0.40 pg/ml) or HSLC-EVs (TNFα 0.08 ± 0.01 and IL-10 1.45 ± 0.28 pg/ml) treatment. The SHAM group had also similar BAL concentration of TNFα and IL-10 (0.08 ± 0.03 and 1.25 ± 0.41 pg/ml, respectively) (Fig. 2c, d).

### Histological analysis

**Fig. 3:**
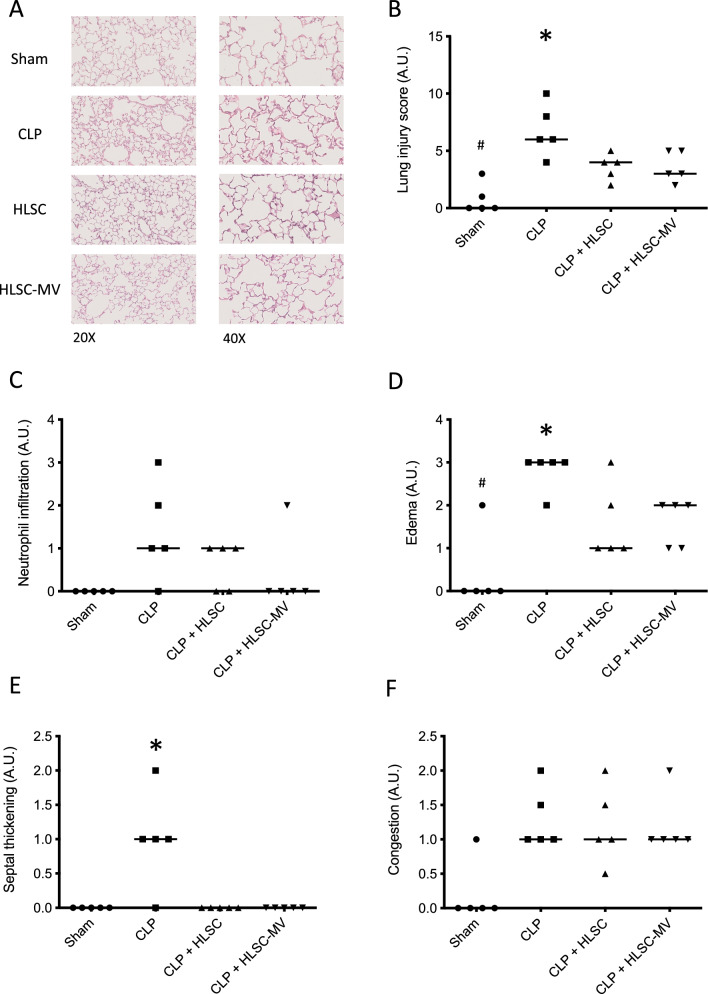
Representative images of lungs’ histologic analysis, stained with haematoxylin and eosin at 20X and 40X magnification from left to right, respectively (**panel A**) and Lung Injury Score (**Panel B to F**) between groups. **Panel B**: total Lung Injury Score; **panel C**: Neutrophile infiltration; **panel D**: Alveolar Oedema; **panel E**: Septal Thickening; **panel F**: Capillary Congestion. Sham: mice subjected to simple median laparotomy without performing CLP; CLP: mice subjected to Caecal Ligation and Puncture treated with IV normal saline; HLSCs: mice subjected to Caecal Ligation and Puncture treated with IV Human Liver Stem Cells; HLSC-EVs: mice subjected to Caecal Ligation and Puncture treated with IV Human Liver Stem Cells’ Extracellular vesicles; FB: mice subjected to Caecal Ligation and Puncture treated with IV Fibroblasts. *p < 0.05 CLP versus all other groups; ^#^*p* < 0.05 Sham versus all other groups. One way ANOVA with Tukey post-hoc test

In mice exposed to CLP and treated with HLSCs or HLSC-EVs, the total LIS was significantly lower compared to the positive control animals after CLP (3.8 ± 1.3 vs. 3.6 ± 1.3 vs. 6.8 ± 2.3 A.U., respectively; *p* < 0.05). Total LIS was lower in the SHAM group (0.8 ± 1.5 A.U) compared to all the other experimental groups (0.8 ± 1.5 A.U vs. all; *p* < 0.05) (Fig. 3b). In particular, the HLSC and HLSC-EVs groups showed significantly lower alveolar oedema (HLSCs 2.0 ± 0.8 A.U. and HLSC-EVs 1.6 ± 0.6 A.U. vs. CLP 2.7 ± 0.5 A.U.; *p* < 0.05—Fig. 3d) and septal thickening (HLSCs 0.0 ± 0.0 A.U. and HLSC-EVs 0.0 ± 0.0 A.U. vs. CLP 1.0 ± 0.7 A.U.; *p* < 0.05—Fig. 3e) compared to the positive controls. However, administration of HLSCs and HLSC-EVs was not able to prevent neutrophil infiltration or capillary congestion induced by CLP (Fig. 3c, f).

### Bone marrow analysis

**Fig. 4:**
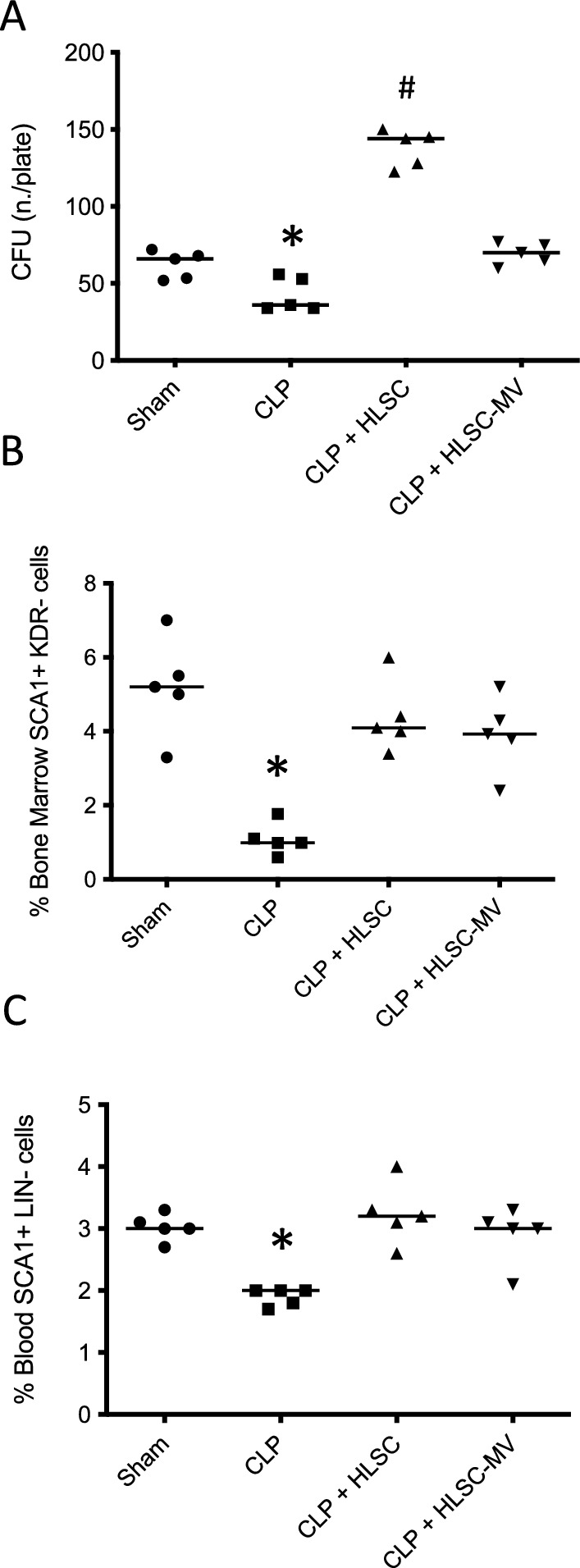
Number of CFC of the bone marrow-derived cells cultured at the concentration of 1*10^4^ in the presence of erythropoietin (10 ng/mL) for 6–7 days (**Panel A**). The colonies formed had been enumerated, identified and counted. Sham: mice subjected to simple median laparotomy without performing CLP; CLP: mice subjected to Caecal Ligation and Puncture treated with IV normal saline; HLSCs: mice subjected to Caecal Ligation and Puncture treated with IV Human Liver Stem Cells; HLSC-EVs: mice subjected to Caecal Ligation and Puncture treated with IV Human Liver Stem Cells’ Extracellular vesicles. Percentage of Sca1 + cells in the bone marrow (**Panel B**) and blood (**Panel C**) evaluated by flow cytometry. *p < 0.05 CLP versus all other groups; ^#^*p* < 0.05 HLSCs versus all other groups. One-way ANOVA with Tukey post-hoc test

CLP resulted in a significant decrease in the total number of CFC from the bone marrow compared to the SHAM (CLP 44 ± 11 CFC vs. SHAM 62 ± 8 CFC; *p* < 0.01). The treatment with 1*10^6^ HLSCs after CLP resulted in higher number of CFC (137 ± 13 CFC) compared to the CLP and compared to the SHAM group (Fig. 4a). The administration of HLSC-EVs after CLP resulted in a higher number of CFC (68 ± 8 CSC) than in the CLP group, similar to those in the SHAM group (Fig. 4a).

Compared to the positive CLP controls, the administration of HLSCs and HLSC-EVs after CLP resulted in a significantly higher number of SCa1 + lineage negative hematopoietic precursors in both bone marrow (*p* < 0.05; Fig. 4b) and matched blood samples (*p* < 0.05; Fig. 4c), reaching similar levels of the SHAM group. Moreover, we didn’t find any significant statistical difference between the groups of animals receiving HLSC-EVs and HLSCs.

## Discussion

This study demonstrates that the administration of HLSCs or HLSC-EVs was effective in improving the outcome in a pre-clinical model of lethal sepsis. The administration of HLSCs or HLSC-EVs in this model resulted in improved bacterial clearance, improved lung function, as well as increment of bone marrow murine hematopoietic progenitors and ultimately enhanced survival.

Previous investigations demonstrated the therapeutic effect of cell-based treatment in animal models of sepsis [5, 14–16]. As highlighted in the systematic review and meta-analysis by Shujun Yang et al. [39], only 13 studies explored the therapeutic effect of stem cell-derived EVs in a clinically relevant model of septic shock, such as CLP. Of these studies, only two employed unfractionated EVs, [40, 41] with just one specifically utilizing MSC-derived EVs [40] rather than exosomes and none of them used HLSC derived EVs. The CLP model is considered the gold standard for sepsis research [42], because it narrows the gap between experimental and clinical conditions, compared to other models of sepsis, such as endotoxin or bacteria administration [43]. CLP more accurately reproduces the time course and the complexity of the pathophysiological changes, which occur during sepsis and septic shock in a living organism after the original insult, with a persistent release of bacteria in blood, conditioning a protracted and severe disease with impact on survival [44]. In our study, we targeted a model of lethal CLP-induced sepsis, which caused mortality in all the positive control animals (100% mortality) within 48 h. This approach aimed to enhance the statistical power of our study by identifying a treatment effect while minimizing the number of experimental animals required, thus serving as a proof of concept. Indeed, treatment with HLSCs or HLSC-EVs 6 h after CLP resulted in a significant increase in survival with an effect size of ~ 20% (Fig. 1d). The positive controls in our model undergoing CLP had a high total bacterial load in peripheral blood, which was significantly reduced after HLSCs or HLSC-EVs administration (Fig. 1e). This is consistent with previous findings both from rodent models of CLP-induced sepsis [16, 45–48] and *E. coli* induced pneumonia [49], in which treatment with MSCs was associated with reduced bacterial count in the blood [16, 45], spleen [15] and lung tissue. [49] Interestingly, MSC-derived EVs showed the same activity as the cells themselves. Furthermore, Monsel et al. showed that in vitro treatment of human monocytes with MSC-EVs increased phagocytosis against bacteria, reducing bacterial count, thereby suggesting a potential mechanism for the beneficial effect of cell-derived EV therapy. [49]

Distant organ damage ultimately leading multiorgan failure and death in septic shock has been the focus of several studies investigating the complex pathophysiology of this condition and potential identification of therapeutic targets [50]. In this regard, MSCs, MSC-EVs and exosomes have been shown to improve kidney injury [15, 16, 47] and oedema, reducing diffuse alveolar damage and liver congestion [51], as well as mitigate liver failure [16, 52] after CLP.

In our model of CLP-induced lethal sepsis, we investigated the effect of the treatment with HLSCs or HLSC-EVs on two major organs involved in the pathogenesis of sepsis, such as lung and bone marrow. In contrast to the untreated CLP positive controls, both experimental groups treated with HLSCs or HLSC-EVs exhibited significantly lower histological lung injury scores following CLP (Fig. 2a). This is consistent with the findings of a previous study from Blanco et al. in which bone marrow-derived EVs were able to mitigate histological diffuse alveolar damage compared to controls after CLP in mice [51]. In addition, in our study, we showed that HLSC-EVs were as effective as HLSCs in preserving the compliance of the respiratory system (Fig. 2a) and the function of the alveolar–epithelial barrier after CLP. The latter effect was demonstrated in our findings by the significantly lower amount of total proteins in the BAL in treated animals compared to CLP positive controls (Fig. 2b). In our model, the therapeutic effect of HLSCs or HLSC-EVs on the lung was not mediated by anti-inflammatory mechanisms. Indeed, administration of HLSCs and HLSC-EVs yielded no change in the BAL concentrations of inflammatory mediators such as TNFα and IL-10 (Fig. 2c, d). Moreover, histological analysis revealed no significant alteration in neutrophil lung infiltration in mice treated with HLSCs or HLSC-EVs following CLP (Fig. 3c). Interestingly, BAL cytokines concentrations were comparable between the CLP group and the sham controls. These observations align with previous findings indicating lower levels of pro-inflammatory cytokines in plasma and BAL, as well as reduced neutrophil infiltration in the lungs of murine CLP models compared to those induced by intra-tracheal endotoxin administration [53, 54]. Other potential mechanisms may contribute to the increased alveolar–epithelial barrier permeability after CLP in our model, including the increase of cellular apoptosis [55] and the increase of the pulmonary hydrostatic pressure due to sepsis-induced acute cardiomyopathy [56, 57]. However, these hypotheses remain speculative and warrant investigation in future studies. Moreover, in an ex vivo experimental model of lung ischemia and reperfusion injury, which involves some of the biological mechanisms observed in sepsis [58–60], the inhibition of lung cells apoptosis resulted in reduced oedema formation and lung mechanic improvement with no effect on cytokine release and neutrophil infiltration [61].

The bone marrow is a crucial organ in the regulation of the response of the immune system to insults caused by infective micro-organisms, as reservoir of multipotent hemopoietic progenitors and stromal cells providing the appropriate generation and release into the systemic circulation of cells accountable for the innate and adaptive immunity. Concurrently, the bone marrow can also be a target of the biological mechanisms triggered during sepsis, with consequent potential impairment and dysfunction [9, 62, 63]. Inflammatory insults and infection can trigger the activation of the so called “emergency myelopoiesis”, to release mature myeloid cells, such as neutrophils and monocytes [64]. Zhang et al. reported in a murine model that granulocyte–macrophage CFUs were significantly increased in the bone marrow after endotoxin administration [65]. The same authors demonstrated that bacteraemia in mice, caused by *E. coli* iv injection, resulted in an expansion of a committed hematopoietic cell pool (c-kit^+^/Sca1^+^) in the bone marrow with a significant increase in granulocyte/macrophage CFU activity [66]. However, this emergency myeloid response might lead to reduced functionality, self-renewal and differentiation potential of the hematopoietic compartment [64]. This has been confirmed by Rodriguez et al. in a murine model of lethal sepsis induced by *Pseudomonas aeruginosa* inoculation, in which the expansion of the hematopoietic stem cells in the bone marrow was associated with their impairment in terms of myeloid differentiation and production [67]. Similarly, Skirecki et al. showed that early hematopoietic stem and progenitor cell proliferation increased while committed progenitor production decreased in the bone marrow of humanized mice, which underwent either CLP sepsis or endotoxemia [68].

The administration of exogenous hematopoietic stem cells has been proposed to restore adequate myelopoiesis and improve survival after septic insults. Morales-Mantilla et al. showed in a murine model of gram-positive bacteria-induced sepsis that the administration of hematopoietic stem and progenitor cells (HSPCs), taken from naïve donor mice, trigger a robust myeloid response, together with hematopoietic stem cells restoring in the bone marrow without direct engrafting [69]. These results confirmed the detrimental effect of sepsis on the bone marrow and demonstrated, as proof of concept, that this injury is responsive to cell-based treatment. However, the proposed therapeutic approach, using progenitor cells from genetically identical healthy subjects, cannot be translated into clinical practise. Our study demonstrated for the first time that the administration of exogenous clinically available stromal cells or their derivatives, such HLSCs and HLSC-EVs, in pre-clinical models of sepsis resulted in the preservation of adequate haematopoiesis in the murine bone marrow and improved survival compared to positive controls (Fig. 4). This is confirmed by the detection with flow cytometry of murine pluripotent hematopoietic stem cells both in the bone marrow (Fig. 4b) and in blood (Fig. 4c). This may be ascribed to the complex cell-to-cell interaction between the administered stem cells on the native hematopoietic murine precursor in the bone marrow, which is known to be mediated via adhesion molecules and paracrine signalling, being cytokines and EVs the potential vehicles of this last mechanism [70–72]. Extracellular vesicles in particular are known to deliver specific mRNA to the target bone marrow cells [21, 73]. The administration of HLSCs or their EVs was able to preserve the native bone marrow replicative potential. This is consistent with the concomitant reduction in bacterial load in the treated mice and the consequent improved survival. In this regard, HLSC-EVs showed similar potential compared to the whole HLSCs, allowing us to speculate that EV-mediated paracrine signalling to bone marrow cells is a key mechanism in our model. The therapeutic equivalence between HLSCs and their EVs is of relevant clinical importance because EVs have a safer profile compared to HSLCs as they are stable for long term storage avoiding the use of carcinogenic cryoprotective agents, together with an intrinsic inability to induce tumours. Moreover, EVs have a lower probability of triggering an immune response and can effectively reach the target tissues, as they easily pass biological barriers [74, 75].

Our study showed the therapeutic potential of HLSCs and their EVs in a clinically relevant model of lethal sepsis. We did not evaluate whether similar effects were achieved with different types of stromal cells, including mesenchymal stem cells, decidual stromal cells, umbilical cord-derived stem cells, used in other models of sepsis [5]. However, few characteristics of the HSLCs may offer specific benefits for their potential therapeutic application. Clinical grade HSLCs are isolated from organ donor liver fragments following Good Manufacturing Practice standards and showed great proliferative potential with telomere stability until the 17th culture passage, indicating a good resistance to senescence and stability [24, 25]. Moreover, as mentioned before, HSLCs can inhibit *T*-cell proliferation to a greater extent than mesenchymal stem cells and, in contrast to MSCs, do not induce NK cell degranulation [31]. This aspect might be clinically relevant, because the detrimental role of NK cells is well documented in both animal models and human studies and their activation is associated with worse survival [76, 77].

This study has some limitations. First, we did not perform a dose–response curve with different numbers of HLSCs or HLSC-EVs: the dose administered to the treatment groups was chosen after few pilot experiments with the endpoint of obtaining a consistent number of cells and EVs (data not shown). Second, the choice to administer the treatment 6 h after CLP and to sacrifice the animals 24 h after CLP for organ analysis was discretional and based on the time estimated during the pilot survival experiments for the intervention to be effective when administered after the insult with enough lag before the potential occurrence of the lethal outcome. Third, we did not perform a direct analysis of the potential specific beneficial cellular mechanisms of the HLSCs or HLSC-EVs involving the interaction with *T*-cells and NKs; however, HLSCs are known to effectively inhibit *T*-cell proliferation without inducing NK cell degranulation [31]. Fourth, the signalling pathways mediating the protective effects of HLSCs and their EVs on lung damage and oedema have not been elucidated. Similarly, the mechanism by which therapy with this specific cell lineage was able to improve myelopoiesis has not been explored. Fifth, our study did not include a control group treated with antibiotics, which are the gold standard for the treatment of sepsis and septic shock. Sixth, we administer human-derived cells in mice, raising the risk of rejection due to immune incompatibility between the donor and the recipient. However, there is a large number of published studies investigating the effect of human-derived cell therapy in animal models. Moreover, Bruno et al. demonstrated that HLSCs appear to have greater protection against allogeneic NK cell lysis compared to MSCs [31]. Furthermore, the use of ultracentrifugation (UC) poses challenges for clinical translation compared to techniques more compatible with large-scale, clinical-grade EV production, such as Tangential Flow Filtration (TFF). However, UC provided a sufficient concentration of vesicles for our specific experimental setup, given the small volumes required for administration in mice. Last, we studied the potential therapeutic effect of HLSCs or HLSC-EVs only in a mouse model of CLP-induced lethal sepsis and not in models of sepsis with less degree of severity. However, this study aimed to provide a proof of concept that can be further explored in future investigations.

## Conclusions

The treatment of experimental lethal sepsis with HLSCs or HLSC-derived EVs proves effective in reducing bacteraemia, improving lung function, alleviating oedema, as well as restoring bone marrow function, and ultimately reducing mortality rates. Further studies are needed to better define the cellular mechanisms driving this therapeutic effect and ascertain its limitations. Our study serves as foundational groundwork for subsequent evaluation of this promising cell-based therapy in a clinical settings.

## Supplementary Information

Additional file 1. Fig. S1.

## Data Availability

The datasets used and/or analysed during the current study are available from the corresponding author on reasonable request.

## Abbreviations

ARDS: Acute respiratory distress syndrome (ARDS)
MSCs: Mesenchymal stromal cells (MSCs)
IL-6: Interleukin 6
IL-8: Interleukin 8
MCP-1: Monocyte Chemoattractant Protein-1
HLA: Human Leukocyte Antigen
NK: Natural killer
EVs: Extracellular vesicles
MSC-EVs: MSC-released EVs
HLSCs: Human liver stem cells
AKI: Acute kidney injury
NHDF: Primary normal Human Dermal Fibroblasts
CLP: Caecal ligation and puncture
Pi: Inspiratory pressure
PEEP: Positive end-expiratory pressure
E: Lung elastance
BAL: Broncho-alveolar lavage
TNF-α: Tumour necrosis factor-α
IL-10: Interleukin-10
LIS: Lung injury score
CFC: Colony-forming cells

## References

[1] Rudd KE, Johnson SC, Agesa KM et al (2020) Global, regional, and national sepsis incidence and mortality, 1990–2017: analysis for the Global Burden of Disease Study. Lancet Lond Engl 395:200–211. 10.1016/S0140-6736(19)32989-7 10.1016/S0140-6736(19)32989-7 PMC6970225 31954465

[2] Shankar-Hari M, Phillips GS, Levy ML et al (2016) Developing a new definition and assessing new clinical criteria for septic shock. JAMA 315:775–787. 10.1001/jama.2016.0289 26903336 10.1001/jama.2016.0289 PMC4910392

[3] Vincent J-L, Jones G, David S et al (2019) Frequency and mortality of septic shock in Europe and North America: a systematic review and meta-analysis. Crit Care 23:196. 10.1186/s13054-019-2478-6 31151462 10.1186/s13054-019-2478-6 PMC6545004

[4] Kwok AJ, Allcock A, Ferreira RC et al (2023) Neutrophils and emergency granulopoiesis drive immune suppression and an extreme response endotype during sepsis. Nat Immunol 24:767–779. 10.1038/s41590-023-01490-5 37095375 10.1038/s41590-023-01490-5

[5] Johnson CL, Soeder Y, Dahlke MH (2017) Concise review: mesenchymal stromal cell-based approaches for the treatment of acute respiratory distress and sepsis syndromes. Stem Cells Transl Med 6:1141–1151. 10.1002/sctm.16-0415 28186706 10.1002/sctm.16-0415 PMC5442840

[6] Singer M, Deutschman CS, Seymour CW et al (2016) The third international consensus definitions for sepsis and septic shock (Sepsis-3). JAMA 315:801. 10.1001/jama.2016.0287 26903338 10.1001/jama.2016.0287 PMC4968574

[7] Ghimire S, Ravi S, Budhathoki R et al (2021) Current understanding and future implications of sepsis-induced thrombocytopenia. Eur J Haematol 106:301–305. 10.1111/ejh.13549 33191517 10.1111/ejh.13549

[8] Loftus TJ, Mohr AM, Moldawer LL (2018) Dysregulated myelopoiesis and hematopoietic function following acute physiologic insult. Curr Opin Hematol 25:37–43. 10.1097/MOH.0000000000000395 29035909 10.1097/MOH.0000000000000395 PMC5733709

[9] Hotchkiss RS, Monneret G, Payen D (2013) Sepsis-induced immunosuppression: from cellular dysfunctions to immunotherapy. Nat Rev Immunol 13:862–874. 10.1038/nri3552 24232462 10.1038/nri3552 PMC4077177

[10] Liu VX, Fielding-Singh V, Greene JD et al (2017) The timing of early antibiotics and hospital mortality in sepsis. Am J Respir Crit Care Med 196:856–863. 10.1164/rccm.201609-1848OC 28345952 10.1164/rccm.201609-1848OC PMC5649973

[11] Rhodes A, Evans LE, Alhazzani W et al (2017) Surviving sepsis campaign: international guidelines for management of sepsis and septic shock: 2016. Crit Care Med 45:486–552. 10.1097/CCM.0000000000002255 28098591 10.1097/CCM.0000000000002255

[12] Seymour CW, Gesten F, Prescott HC et al (2017) Time to treatment and mortality during mandated emergency care for sepsis. N Engl J Med 376:2235–2244. 10.1056/NEJMoa1703058 28528569 10.1056/NEJMoa1703058 PMC5538258

[13] Laroye C, Gibot S, Huselstein C, Bensoussan D (2020) Mesenchymal stromal cells for sepsis and septic shock: lessons for treatment of COVID-19. Stem Cells Transl Med 9:1488–1494. 10.1002/sctm.20-0239 32808462 10.1002/sctm.20-0239 PMC7461462

[14] Lalu MM, Sullivan KJ, Mei SH et al (2016) Evaluating mesenchymal stem cell therapy for sepsis with preclinical meta-analyses prior to initiating a first-in-human trial. Elife. 10.7554/eLife.17850 27870924 10.7554/eLife.17850 PMC5153252

[15] Mei SHJ, Haitsma JJ, Dos Santos CC et al (2010) Mesenchymal stem cells reduce inflammation while enhancing bacterial clearance and improving survival in sepsis. Am J Respir Crit Care Med 182:1047–1057. 10.1164/rccm.201001-0010OC 20558630 10.1164/rccm.201001-0010OC

[16] Németh K, Leelahavanichkul A, Yuen PST et al (2009) Bone marrow stromal cells attenuate sepsis via prostaglandin E2–dependent reprogramming of host macrophages to increase their interleukin-10 production. Nat Med 15:42–49. 10.1038/nm.1905 19098906 10.1038/nm.1905 PMC2706487

[17] McIntyre LA, Stewart DJ, Mei SHJ et al (2018) Cellular immunotherapy for septic shock. A Phase I clinical trial. Am J Respir Crit Care Med 197:337–347. 10.1164/rccm.201705-1006OC 28960096 10.1164/rccm.201705-1006OC

[18] Schlosser K, Wang J-P, Dos Santos C et al (2019) Effects of Mesenchymal Stem Cell Treatment on Systemic Cytokine Levels in a Phase 1 Dose Escalation Safety Trial of Septic Shock Patients. Crit Care Med 47:918–925. 10.1097/CCM.0000000000003657 30720538 10.1097/CCM.0000000000003657 PMC6629173

[19] Le Blanc K, Davies LC (2015) Mesenchymal stromal cells and the innate immune response. Immunol Lett 168:140–146. 10.1016/j.imlet.2015.05.004 25982165 10.1016/j.imlet.2015.05.004

[20] Abbasi B, Shamsasenjan K, Ahmadi M et al (2022) Mesenchymal stem cells and natural killer cells interaction mechanisms and potential clinical applications. Stem Cell Res Ther 13:97. 10.1186/s13287-022-02777-4 35255980 10.1186/s13287-022-02777-4 PMC8900412

[21] Quaglia M, Fanelli V, Merlotti G et al (2022) Dual role of extracellular vesicles in sepsis-associated kidney and lung injury. Biomedicines 10:2448. 10.3390/biomedicines10102448 36289710 10.3390/biomedicines10102448 PMC9598620

[22] Cheng Y, Cao X, Qin L (2020) Mesenchymal stem cell-derived extracellular vesicles: a novel cell-free therapy for sepsis. Front Immunol 11:647. 10.3389/fimmu.2020.00647 32373121 10.3389/fimmu.2020.00647 PMC7186296

[23] Herrera MB, Bruno S, Buttiglieri S et al (2006) Isolation and Characterization of a Stem Cell Population from Adult Human Liver. Stem Cells 24:2840–2850. 10.1634/stemcells.2006-0114 16945998 10.1634/stemcells.2006-0114

[24] Bruno S, Herrera Sanchez MB, Chiabotto G et al (2021) Human liver stem cells: a liver-derived mesenchymal stromal cell-like population with pro-regenerative properties. Front Cell Dev Biol 9:644088. 10.3389/fcell.2021.644088 33981703 10.3389/fcell.2021.644088 PMC8107725

[25] Bruno S, Herrera Sanchez MB, Pasquino C et al (2019) Human liver-derived stem cells improve fibrosis and inflammation associated with nonalcoholic steatohepatitis. Stem Cells Int 2019:6351091. 10.1155/2019/6351091 31281379 10.1155/2019/6351091 PMC6589210

[26] Herrera MB, Fonsato V, Bruno S et al (2013) Human liver stem cells improve liver injury in a model of fulminant liver failure. Hepatology 57:311–319. 10.1002/hep.25986 22829291 10.1002/hep.25986

[27] Fonsato V, Lena MD, Tritta S, et al (2018) Human liver stem cell-derived extracellular vesicles enhance cancer stem cell sensitivity to tyrosine kinase inhibitors through Akt/mTOR/PTEN combined modulation. Oncotarget 9:36151. 10.18632/oncotarget.26319 10.18632/oncotarget.26319 PMC6281417 30546834

[28] Fanelli V, Puntorieri V, Assenzio B et al (2010) Pulmonary-derived phosphoinositide 3-kinase gamma (PI3Kγ) contributes to ventilator-induced lung injury and edema. Intensive Care Med 36:1935–1945. 10.1007/s00134-010-2018-y 20721532 10.1007/s00134-010-2018-y

[29] Bruno S, Chiabotto G, Cedrino M et al (2022) Extracellular vesicles derived from human liver stem cells attenuate chronic kidney disease development in an in vivo experimental model of renal ischemia and reperfusion injury. Int J Mol Sci 23:1485. 10.3390/ijms23031485 35163409 10.3390/ijms23031485 PMC8835844

[30] Sanchez MBH, Bruno S, Grange C et al (2014) Human liver stem cells and derived extracellular vesicles improve recovery in a murine model of acute kidney injury. Stem Cell Res Ther 5:124. 10.1186/scrt514 25384729 10.1186/scrt514 PMC4446072

[31] Bruno S, Grange C, Tapparo M et al (2016) Human liver stem cells suppress T-Cell Proliferation, NK Activity, and dendritic cell differentiation. Stem Cells Int 2016:1–14. 10.1155/2016/8468549 10.1155/2016/8468549 PMC4834412 27127520

[32] Grange C, Tapparo M, Bruno S et al (2014) Biodistribution of mesenchymal stem cell-derived extracellular vesicles in a model of acute kidney injury monitored by optical imaging. Int J Mol Med 33:1055–1063. 10.3892/ijmm.2014.1663 24573178 10.3892/ijmm.2014.1663 PMC4020482

[33] Koliha N, Wiencek Y, Heider U et al (2016) A novel multiplex bead-based platform highlights the diversity of extracellular vesicles. J Extracell Vesicles. 10.3402/jev.v5.29975 26901056 10.3402/jev.v5.29975 PMC4762227

[34] Rittirsch D, Huber-Lang MS, Flierl MA, Ward PA (2009) Immunodesign of experimental sepsis by cecal ligation and puncture. Nat Protoc 4:31–36. 10.1038/nprot.2008.214 19131954 10.1038/nprot.2008.214 PMC2754226

[35] Bruno S, Pasquino C, Herrera Sanchez MB et al (2020) HLSC-derived extracellular vesicles attenuate liver fibrosis and inflammation in a murine model of non-alcoholic steatohepatitis. Mol Ther J Am Soc Gene Ther 28:479–489. 10.1016/j.ymthe.2019.10.016 10.1016/j.ymthe.2019.10.016 PMC7001005 31757759

[36] Stenqvist O (2003) Practical assessment of respiratory mechanics. BJA Br J Anaesth 91:92–105. 10.1093/bja/aeg141 12821569 10.1093/bja/aeg141

[37] Fanelli V, Mascia L, Puntorieri V et al (2009) Pulmonary atelectasis during low stretch ventilation: “Open lung” versus “lung rest” strategy*. Crit Care Med 37:1046. 10.1097/CCM.0b013e3181968e7e 19237916 10.1097/CCM.0b013e3181968e7e

[38] Godshall CJ, Scott MJ, Peyton JC et al (2002) Genetic background determines susceptibility during murine septic peritonitis. J Surg Res 102:45–49. 10.1006/jsre.2001.6319 11792151 10.1006/jsre.2001.6319

[39] Yang S, Zhang K, Hou J et al (2023) Protective properties of extracellular vesicles in sepsis models: a systematic review and meta-analysis of preclinical studies. J Transl Med 21:262. 10.1186/s12967-023-04121-7 37069645 10.1186/s12967-023-04121-7 PMC10108460

[40] Chen J, Li C, Liang Z et al (2021) Human mesenchymal stromal cells small extracellular vesicles attenuate sepsis-induced acute lung injury in a mouse model: the role of oxidative stress and the mitogen-activated protein kinase/nuclear factor kappa B pathway. Cytotherapy 23:918–930. 10.1016/j.jcyt.2021.05.009 34272174 10.1016/j.jcyt.2021.05.009

[41] Gao Y, Jin H, Tan H et al (2022) Erythrocyte-derived extracellular vesicles aggravate inflammation by promoting the proinflammatory macrophage phenotype through TLR4–MyD88–NF-κB–MAPK pathway. J Leukoc Biol 112:693–706. 10.1002/JLB.3A0821-451RR 35411633 10.1002/JLB.3A0821-451RR

[42] Buras JA, Holzmann B, Sitkovsky M (2005) Animal Models of sepsis: setting the stage. Nat Rev Drug Discov 4:854–865. 10.1038/nrd1854 16224456 10.1038/nrd1854

[43] Dejager L, Pinheiro I, Dejonckheere E, Libert C (2011) Cecal ligation and puncture: The gold standard model for polymicrobial sepsis? Trends Microbiol 19:198–208. 10.1016/j.tim.2011.01.001 21296575 10.1016/j.tim.2011.01.001

[44] Seemann S, Zohles F, Lupp A (2017) Comprehensive comparison of three different animal models for systemic inflammation. J Biomed Sci 24:60. 10.1186/s12929-017-0370-8 28836970 10.1186/s12929-017-0370-8 PMC5569462

[45] Alcayaga-Miranda F, Cuenca J, Martin A et al (2015) Combination therapy of menstrual derived mesenchymal stem cells and antibiotics ameliorates survival in sepsis. Stem Cell Res Ther. 10.1186/s13287-015-0192-0 26474552 10.1186/s13287-015-0192-0 PMC4609164

[46] Gonzalez H, Keane C, Masterson CH et al (2020) Umbilical Cord-Derived CD362+ Mesenchymal Stromal Cells Attenuate Polymicrobial Sepsis Induced by Caecal Ligation and Puncture. Int J Mol Sci 21:8270. 10.3390/ijms21218270 33158246 10.3390/ijms21218270 PMC7672591

[47] Luo C, Zhang F, Zhang L et al (2014) Mesenchymal stem cells ameliorate sepsis-associated acute kidney injury in mice. Shock 41:123–129. 10.1097/SHK.0000000000000080 24169208 10.1097/SHK.0000000000000080

[48] Luo C, Luo F, Che L et al (2023) Mesenchymal stem cells protect against sepsis-associated acute kidney injury by inducing Gal-9/Tim-3 to remodel immune homeostasis. Ren Fail 45:2187229. 10.1080/0886022X.2023.2187229 36883358 10.1080/0886022X.2023.2187229 PMC10013538

[49] Monsel A, Zhu Y, Gennai S et al (2015) Therapeutic Effects of Human Mesenchymal Stem Cell–derived Microvesicles in Severe Pneumonia in Mice. Am J Respir Crit Care Med 192:324–336 26067592 10.1164/rccm.201410-1765OC PMC4584251

[50] Pinsky MR, Vincent J-L, Deviere J et al (1993) Serum cytokine levels in human septic shock: relation to multiple-system organ failure and mortality. Chest 103:565–575. 10.1378/chest.103.2.565 8432155 10.1378/chest.103.2.565

[51] Blanco NG, Machado NM, Castro LL et al (2023) Extracellular vesicles from different sources of mesenchymal stromal cells have distinct effects on lung and distal organs in experimental sepsis. Int J Mol Sci 24:8234. 10.3390/ijms24098234 37175936 10.3390/ijms24098234 PMC10179270

[52] Cai J, Tang D, Hao X et al (2023) Mesenchymal stem cell-derived exosome alleviates sepsis- associated acute liver injury by suppressing MALAT1 through microRNA-26a-5p: an innovative immunopharmacological intervention and therapeutic approach for sepsis. Front Immunol 14:1157793. 10.3389/fimmu.2023.1157793 37398640 10.3389/fimmu.2023.1157793 PMC10310917

[53] Chimenti L, Morales-Quinteros L, Puig F et al (2020) Comparison of direct and indirect models of early induced acute lung injury. Intensive Care Med Exp 8:62. 10.1186/s40635-020-00350-y 33336290 10.1186/s40635-020-00350-y PMC7746791

[54] Remick DG, Newcomb DE, Bolgos GL, Call DR (2000) Comparison of the mortality and inflammatory response of two models of sepsis: lipopolysaccharide vs. cecal ligation and puncture. Shock Augusta Ga 13:110–116. 10.1097/00024382-200013020-00004 10670840 10.1097/00024382-200013020-00004

[55] Hiramatsu M, Hotchkiss RS, Karl IE, Buchman TG (1997) Cecal ligation and puncture (CLP) induces apoptosis in thymus, spleen, lung, and gut by an endotoxin and TNF-independent pathway. Shock Augusta Ga 7:247–253. 10.1097/00024382-199704000-00002 9110409 10.1097/00024382-199704000-00002

[56] Carbone F, Liberale L, Preda A et al (2022) Septic cardiomyopathy: from pathophysiology to the clinical setting. Cells 11:2833. 10.3390/cells11182833 36139408 10.3390/cells11182833 PMC9496713

[57] Hobai IA, Edgecomb J, LaBarge K, Colucci WS (2015) Dysregulation of intracellular calcium transporters in animal models of sepsis induced cardiomyopathy. Shock Augusta Ga 43:3–15. 10.1097/SHK.0000000000000261 25186837 10.1097/SHK.0000000000000261 PMC4269564

[58] Matsuda A, Jacob A, Wu R et al (2011) Milk Fat Globule-EGF Factor VIII in Sepsis and Ischemia-Reperfusion Injury. Mol Med 17:126–133. 10.2119/molmed.2010.00135 20882259 10.2119/molmed.2010.00135 PMC3022991

[59] Okutani D, Lodyga M, Han B, Liu M (2006) Src protein tyrosine kinase family and acute inflammatory responses. Am J Physiol-Lung Cell Mol Physiol 291:L129–L141. 10.1152/ajplung.00261.2005 16581827 10.1152/ajplung.00261.2005

[60] Pendyala S, Usatyuk PV, Gorshkova IA et al (2009) Regulation of NADPH oxidase in vascular endothelium: the role of phospholipases, protein kinases, and cytoskeletal proteins. Antioxid Redox Signal 11:841–860. 10.1089/ars.2008.2231 18828698 10.1089/ars.2008.2231 PMC2850292

[61] Del Sorbo L, Costamagna A, Muraca G, et al (2016) Intratracheal Administration of Small Interfering RNA Targeting Fas Reduces Lung Ischemia-Reperfusion Injury*. Crit Care Med 44:e604–e613. 10.1097/CCM.0000000000001601 10.1097/CCM.0000000000001601 26963318

[62] Annane D, Bellissant E, Cavaillon J-M (2005) Septic shock. The Lancet 365:63–78. 10.1016/S0140-6736(04)17667-8 10.1016/S0140-6736(04)17667-8 15639681

[63] Venet F, Monneret G (2018) Advances in the understanding and treatment of sepsis-induced immunosuppression. Nat Rev Nephrol 14:121–137. 10.1038/nrneph.2017.165 29225343 10.1038/nrneph.2017.165

[64] Kelly LS, Darden DB, Fenner BP et al (2021) The hematopoietic stem/progenitor cell response to hemorrhage, injury, and sepsis: a review of pathophysiology. Shock Augusta Ga 56:30–41. 10.1097/SHK.0000000000001699 33234838 10.1097/SHK.0000000000001699 PMC8141062

[65] Zhang P, Quinton LJ, Gamble L et al (2005) The granulopoietic cytokine response and enhancement of granulopoiesis in mice during endotoxemia. Shock Augusta Ga 23:344–352. 10.1097/01.shk.0000158960.93832.de 15803058 10.1097/01.shk.0000158960.93832.de

[66] Zhang P, Nelson S, Bagby GJ et al (2008) The Lineage-c-kit+Sca-1+ Cell Response to Escherichia Coli Bacteremia in Balb/c Mice. Stem Cells Dayt Ohio 26:1778–1786. 10.1634/stemcells.2007-1027 10.1634/stemcells.2007-1027 PMC2731662 18483422

[67] Rodriguez S, Chora A, Goumnerov B et al (2009) Dysfunctional expansion of hematopoietic stem cells and block of myeloid differentiation in lethal sepsis. Blood 114:4064–4076. 10.1182/blood-2009-04-214916 19696201 10.1182/blood-2009-04-214916 PMC2774548

[68] Skirecki T, Kawiak J, Machaj E et al (2015) Early severe impairment of hematopoietic stem and progenitor cells from the bone marrow caused by CLP sepsis and endotoxemia in a humanized mice model. Stem Cell Res Ther. 10.1186/s13287-015-0135-9 26272069 10.1186/s13287-015-0135-9 PMC4536694

[69] Morales-Mantilla DE, Kain B, Le D et al (2022) Hematopoietic stem and progenitor cells improve survival from sepsis by boosting immunomodulatory cells. Elife 11:e74561. 10.7554/eLife.74561 35166205 10.7554/eLife.74561 PMC8846591

[70] Kulkarni R, Kale V (2020) Physiological cues involved in the regulation of adhesion mechanisms in hematopoietic stem cell fate decision. Front Cell Dev Biol 8:611. 10.3389/fcell.2020.00611 32754597 10.3389/fcell.2020.00611 PMC7366553

[71] Li T, Wu Y (2011) Paracrine molecules of mesenchymal stem cells for hematopoietic stem cell niche. Bone Marrow Res 2011:353878. 10.1155/2011/353878 22046560 10.1155/2011/353878 PMC3196250

[72] Pendse S, Kale V, Vaidya A (2022) The Intercellular Communication Between Mesenchymal Stromal Cells and Hematopoietic Stem Cells Critically Depends on NF-κB Signalling in the Mesenchymal Stromal Cells. Stem Cell Rev Rep 18:2458–2473. 10.1007/s12015-022-10364-6 35347654 10.1007/s12015-022-10364-6

[73] Aliotta JM, Pereira M, Johnson KW et al (2010) Microvesicle entry into marrow cells mediates tissue-specific changes in mRNA by direct delivery of mRNA and induction of transcription. Exp Hematol 38:233–245. 10.1016/j.exphem.2010.01.002 20079801 10.1016/j.exphem.2010.01.002 PMC2829939

[74] Vader P, Mol EA, Pasterkamp G, Schiffelers RM (2016) Extracellular vesicles for drug delivery. Adv Drug Deliv Rev 106:148–156. 10.1016/j.addr.2016.02.006 26928656 10.1016/j.addr.2016.02.006

[75] Zheng G, Huang R, Qiu G et al (2018) Mesenchymal stromal cell-derived extracellular vesicles: regenerative and immunomodulatory effects and potential applications in sepsis. Cell Tissue Res 374:1–15. 10.1007/s00441-018-2871-5 29955951 10.1007/s00441-018-2871-5

[76] Barkhausen T, Frerker C, Pütz C et al (2008) Depletion of NK cells in a murine polytrauma model is associated with improved outcome and a modulation of the inflammatory response. Shock 30:401–410. 10.1097/SHK.0b013e31816e2cda 18391857 10.1097/SHK.0b013e31816e2cda

[77] Gogos C, Kotsaki A, Pelekanou A et al (2010) Early alterations of the innate and adaptive immune statuses in sepsis according to the type of underlying infection. Crit Care 14:R96. 10.1186/cc9031 20504311 10.1186/cc9031 PMC2911733

