# Supplementary figures and images for: Human liver stem cells and derived extracellular vesicles protect from sepsis-induced acute lung injury and restore bone marrow myelopoiesis in a murine model of sepsis
Source: Intensive Care Med Exp. 2024 Dec 3;12:111. doi: 10.1186/s40635-024-00701-z (PMC11615238; doi:10.1186/s40635-024-00701-z)

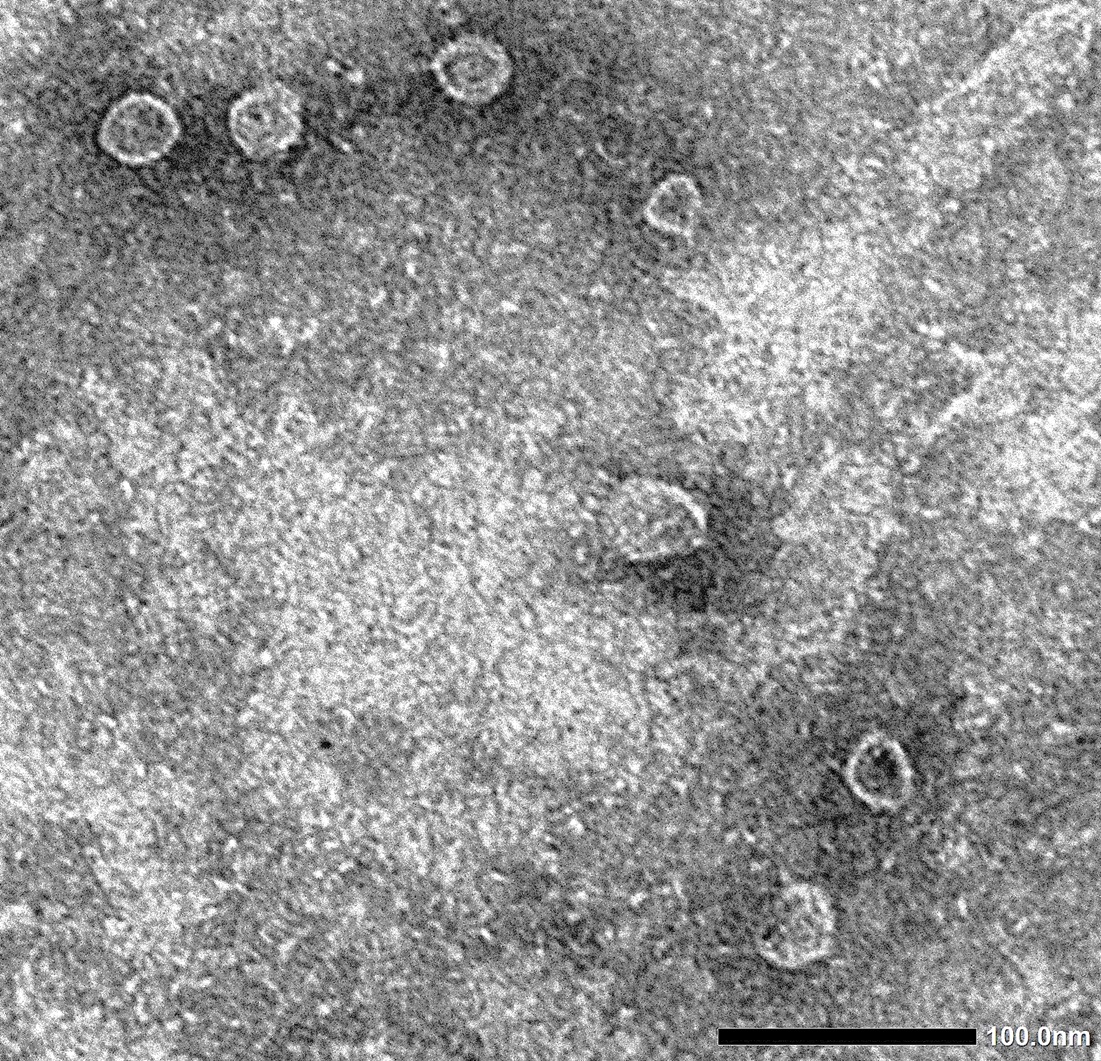

Supplement: Supplementary file 1 — Additional file 1. Fig. S1. [file 40635_2024_701_MOESM1_ESM.jpg]
